# Physicians’ perspectives of palliative care quality in nursing homes: a cross-sectional study

**DOI:** 10.1080/02813432.2026.2742320

**Published:** 2026-10-05

**Authors:** Tuva Sandsdalen, Reidun Hov, Harald Sanaker, Liv Skomakerstuen Ødbehr

**Affiliations:** ^a^ Department of Health and Nursing Sciences, Faculty of Social and Health Sciences, University of Inland Norway, Elverum, Norway; ^b^ Centre for Development of Institutional and Home Care Services, Inland (Hedmark), Hamar Municipality, Norway; ^c^ Municipal Medical Center, Ringsaker Municipality, Brumunddal, Norway

**Keywords:** Palliative care, physicians’ perception, nursing home, quality of healthcare, quality from the patients’ perspective for palliative care (QPP-PC)

## Abstract

**Background:**

Since physicians have primary responsibility for medical treatment, their perspectives of palliative care quality in nursing homes can provide important knowledge for service evaluation and enhancement in this context. However, few studies exist that include their perspectives.

**Objectives:**

The aim was to investigate physicians’ perspectives of the quality of palliative care in Norwegian nursing homes.

**Methods:**

A cross-sectional study conducted between November 2022 and December 2023, which included 77 nursing home physicians (response rate 43.5%) in Norway. Data were collected using a questionnaire comprising questions about demographic variables and perceptions of quality of care, as measured by the short form of the instrument Quality from the Patients’ Perspective for Palliative Care (QPP-PC), adapted for healthcare personnel. Descriptive analyses and Wilcoxon signed rank tests were performed.

**Results:**

Nursing home physicians scored subjective importance of the care significantly higher than their perception of the quality of actual care received in the two dimensions; medical–technical competence and sociocultural atmosphere and for nine out of the 24 items and three out of seven additional symptom-specific questions. A need for improvement was identified in care areas such as symptom relief for pain, depression and anxiety, existential care, understanding patients’ perspective, waiting time, individualized care, cooperation, and continuity.

**Conclusion:**

The results show that provision of high-quality palliative care in nursing homes can be challenging and that the physicians identified several areas for improvement. The results can provide direction for future quality improvement. However, the findings need to be confirmed in a larger sample.

## Background

Palliative care is a multidisciplinary approach that incorporates the prevention and alleviation of physical, psychosocial and spiritual symptoms to improve the quality of life of patients with life-threatening illnesses, regardless of diagnosis [1]. For elderly patients near the end of life in nursing homes (NHs), palliative care involves person-centred and comprehensive care and includes care for dying patients in the last hours, days, or weeks of life [2]. More than half of all deaths in Norway every year occur in NHs [3] Patients in NHs are mostly in poor health and their average lifespan is about two years [3]. Many of the patients have dementia and comorbidities, which complicate care and treatment, and they may need help from their relatives to express their needs to physicians and other healthcare personnel [4–6].

Physicians in NHs (NHP) have the main responsibility for medical treatment of patients [4,5,7]. The NHPs’ expertise is linked to patients’ symptom relief with the aim of improving their quality of life [4]. Physicians in NHs often work with an interdisciplinary team consisting of nurses, nursing assistants, occupational therapists, or other relevant professional groups, who form a care team around the patient. High work pressure and little time for each patient mean that physicians must rely on the collective expertise of the team to provide patients with effective symptom relief and palliative care [8–11]. This means that the joint expertise of healthcare personnel (HCP) is fundamental to quality in end-of-life care [2,12,13].

Quality of care is a complex and multidimensional concept. Perceptions of this concept may differ according to time, culture, levels (individual, organizational, or societal level), and depend on who defines it: patients, relatives, healthcare personnel leaders or policymakers [14,15]. The term care quality in the present study is based on a framework of quality from the patients’ perspective developed by Wilde et al. [14] According to this theoretical framework, care quality is formed through norms, expectations, and experience, and an encounter with a care structure [14]. Care quality can therefore be seen as a measure of a person’s perceptions of the actual care received (the perceived reality) and how important the various care aspects are to that person (the subjective importance of the care aspects) [14].

Previous studies have investigated palliative care quality as perceived by patients and how patients’ experiences of care provided by physicians [16–18]. Patients appreciated physicians providing sufficient information about their illness and their situation, and receiving support from physicians [18]. Furthermore, patients valued settings where physicians would listen to them, and be honest and gentle throughout the dialogue [18]. Another study found that quality in palliative care was described as the physician’s ability to make decisions and their competence in social interactions [13].

Relatively few studies describe physicians’ perspectives on quality in palliative care, especially from the NH context. A recent qualitative study points out that NHPs find that quality in palliative care in nursing homes is about implementing advance care planning, addressing patients’ existential concerns and needs, and safeguarding patients’ autonomy and personal values [19]. Another qualitative study showed that NHPs found that the care quality in NHs was enhanced through comprehensive care plans, a team-based approach, and continuity of care [8].

However, none of these studies examined, in a quantitative manner, how NHPs’ assess the quality of palliative care provided to patients. Physicians’ perspectives and experience of palliative care in this regard may provide important components for service evaluation and enhancement, especially by giving voice to patients in nursing homes that are unable to communicate their views about the care they receive and the importance of this care, due to advanced illness and/or cognitive impairment. The knowledge gained could inform policy-makers, healthcare managers and HCP on how to identify potential areas for improvement. The lack of research in this area is the rationale for this study.

The study aim was to investigate physicians’ perspectives of the quality of palliative care in Norwegian nursing homes.

## Methods

### Design

A cross-sectional study using a questionnaire was conducted. The present study is part of a larger study, which aims to investigate palliative care quality and use of advance care planning in NHs from the perspectives of physicians and patients (PALCARE). The manuscript adheres to the ‘Strengthening the Reporting of Observational Studies in Epidemiology’ (STROBE) reporting guidelines [20] adapted for cross-sectional studies.

### Participants and setting

The present study used purposive sampling to recruit respondents. Respondents were recruited using the following inclusion criteria: Being a medical doctor working full-time or part-time in NHs (NHP). The NHPs were recruited in two ways; through municipal healthcare leaders from municipalities representing all the health regions of Norway and from a network of NHPs in Eastern Norway.

Norwegian NHs are residential care facilities within primary care. They provide both short- and long-term care. Approximately 37,000 people live in NHs in Norway, and three out of four are aged 80 or older [3]. Most of these patients have extensive and often long-term healthcare needs and receive comprehensive inpatient care. Norwegian municipalities organize medical services in NHs either through permanently employed NHPs or by GPs whose practice includes NH services. In 2024, NHPs’ average physician hours per week per nursing home resident was 0.68 at the national level, with a range of 0.53–0.80 between different municipalities [21]. The Norwegian NH context is often characterized as non-specialized in palliative care, where personnel have only basic competence and training in providing palliative care (generalist palliative care) [2]. However, some NHs offer specialized palliative care units or beds and personnel with qualifications in palliative care/medicine, dementia care or cancer care for patients in the late palliative phase. Municipalities in this study were selected to represent densely and sparsely populated areas in all the health regions of Norway and the NHs could be either public or private/non-profit NH organizations, providing long-term and/or short-term care and/or palliative care units or beds.

### Data collection and procedure

The data were collected between November 2022 and December 2023. Information about the study was sent to municipal healthcare leaders. The questionnaire, including instructions on how to fill it out and study information, were sent to NHPs or handed out at a network meeting. Respondents were instructed to return the questionnaire in a sealed envelope.

### Instruments

To measure NHPs’ perspectives of palliative care quality in NHs, the short form of the instrument Quality from the Patients’ Perspective for Palliative Care (QPP-PC) was used [22]. The QPP-PC is based on the theoretical foundation of the validated general instrument Quality from the Patients’ Perspective (QPP) [14,23,24] and it has previously been developed and psychometrically evaluated for the palliative care context [16]. The short version of the instrument has also been adapted and used to measure the perceptions of palliative care quality of HCP (including physicians) on behalf of the patients [25,26]. The short version of the QPP-PC consists of 24 items (20 items within four dimensions and four single items); the dimensions represent palliative care quality, as follows: the medical–technical competence of the caregiver, the physical-technical conditions of the care organization, the identity-oriented approach of the caregivers, and the sociocultural atmosphere of the care organization. The four single items concerned medical care, personal hygiene, spiritual care, and atmosphere in the ward [22]. In this study, seven symptom-specific items from the long version of the QPP-PC [16] were included to measure, in a more precise manner, the most common symptoms that physicians assess and alleviate in palliative care [27,28].

Each of the 24 items of the QPP-PC instrument was measured in two ways: 1) NHPs’ experiences of the care received (perceived reality – PR scale) and 2) how important each aspect of care was perceived by the NHP to be for the patients (subjective importance – SI scale). Items in the PR scale was related to the sentence ‘This is how it is for the patients …’, while those in the SI scale were related to the sentence ‘This is how important this is to the patients …’. The NHP responded to each item on a 4-point (Likert-type) response scale, ranging from 1 (do not agree at all) to 4 (fully agree) for the PR scale and from 1 (of little or no importance) to 4 (of the very highest importance) for the SI scale. The response option N/A (non-applicable) was available for each item. In the identity-oriented approach dimension in the QPP-PC questionnaire, there were also three yes/no items about whether patients had received information about their responsible physician and nurse and their opportunity to participate in their individual care plan.

The questionnaire also contained ten questions about respondents’ characteristics such as age, gender, education, percentage of full-time position, level of education, specialist training/education, work experience in general and in NHs and palliative care, and workload. The final three questions concerned organizational factors, such as size of the NH (number of beds) and availability of specialized palliative care units and/or palliative care beds in the NHs.

The face and content validity of the instrument used in this study were pilot-tested by three experienced NHPs and discussed in the research group, since the instrument had not been previously used by NHPs in this specific care context in Norway. The pilot test included i) comprehensibility of the questionnaire and the instructions for filling it out, ii) the time spent on filling it out, iii) whether the questions covered important aspects of palliative care quality, and iv) whether the pilot testers had suggestions for improvements or inclusion of any missing aspects. The pilot test only led to minor amendments, such as the order of the demographic questions.

### Analysis

IBM SPSS Statistics Data Editor Software, version 23 [29], was used to analyze the data. Descriptive statistics, means and standard deviations were used to describe demographics and perceptions of quality of care.

Due to the relatively small sample size and lack of normal data distribution, Wilcoxon signed rank tests were performed [30] for each QPP-PC dimension on the PR and SI scales and single items, in order to compare the scores on the PR and SI scales. The results of the analyses are described as mean and standard deviation, to be comparable to other studies using this instrument. The mean value was calculated based on the individual participant’s response to the various items in the dimensions of QPP-PC. No cut-off value has been established between high and low QPP-PC PR and SI scores. In line with previous studies, the PR and SI scores are interpreted as high (≥3.50) and low (<3.00) [25,26].

To assess internal consistency of the QPP-PC instrument for this specific sample, the Cronbach’s α analyses were performed for the PR and SI scales at the dimensional level (including single items). The Cronbach’s α values are presented in the description of the QPP-PC and values >0.7 were regarded as desirable [30]. In the present study, Cronbach’s α for the QPP-PC ranged between 0.77 and 0.90 for all dimensions in the two scales, except for physical-technical conditions, where alpha levels were 0.55 and 0.64 for the PR and SI scale, respectively. A *p-*value <0.05 was considered statistically significant for all analyses.

### Ethical approval, considerations and consent to participate

The present study was undertaken in line with the ethical and legal principles of the Declaration of Helsinki and the rules of the EU General Data Protection Regulation and was approved by the Norwegian Agency for Shared Services in Education and Research (SIKT) (ID: 777028) and the local research ethics committee at University of Inland Norway (Ref. No.: 23915412). Approval was not required from the regional ethical research committee to conduct the study (REK) (Ref. No: 502837). All respondents received written information about the study and were informed that participation in the study was voluntary, that they could withdraw their consent at any time without giving a reason and that all personal data would then be deleted. Respondents were also informed that de-identified data would be used for scientific publications. Written, informed consent to participate was obtained from the respondents. Permission was obtained to use the QPP-PC questionnaire.

## Results

### Respondents’ characteristics

A total of 177 NHPs were invited to participate. Of these, 77 returned the questionnaire, giving a response rate of 43.5%. Table 1 shows the characteristics of the respondents included. Most were female and 30–50 years of age. The majority worked in NHs without palliative care units and did not have specialized training/education in palliative care. Of the respondents, 39 had specialist training/further education. Of these 31 as GP, geriatrics (*n* = 4), oncology (*n* = 1) and palliative care (*n* = 3) and 18 were undergoing specialist training. Their work experience varied, but most respondents had relatively long work experience (10–20 years) and experience of palliative care.

**Table 1.** Respondents’ characteristics (*N* = 77).

Variable	N	%	Range	Missing
Age (years)				0
<30	4	5.2		
30–50	41	53.2		
51–70	27	35.1		
>70	5	6.5		
Gender				0
Male	35	45.5		
Female	42	54.5		
Highest level of education				1
Medical doctor	73	94.8		
Medical doctor and PhD	3	3.9		
Percentage of full-time position				1
81–100%	37	48.1		
61–80%	10	13.0		
41–60%	7	9.1		
20–40%	16	20.8		
<20%	6	7.8		
Work experience as a physician (years)				1
<5	6	7.9		
5–10	13	17.1		
11–20	25	32.9		
21–30	13	17.1		
31–40	12	15.8		
>40	7	9.2		
Work experience in palliative care (years)				0
<5	18	23.4		
5–10	24	31.2		
11–20	24	31.2		
21–30	4	5.2		
31–40	5	6.5		
>40	2	2.6		
Availability of palliative care unit in the participant’s workplace (NH)				3
Yes	23	31.1		
No	51	68.9		
Number of palliative care beds in the participant’s workplace (NH)			0–10	0

### Respondents’ perceptions of the quality of care

The respondents’ perceptions of palliative care quality are presented in Table 2. The results are presented as scores for perceptions of palliative care received (PR) and the subjective importance (SI) of the care (how important the NHP perceived that the care area was to patients).

**Table 2.** Comparison of nursing home physicians’ perceptions of perceived reality and subjective importance of care received by dimension, item and single item (*N* = 77).

Dimensions/items/single items	Perceived reality (PR)	Subjective importance (SI)		
	Mean (SD)	Mean (SD)	*n*	*P* *
**Medical–technical competence **	2.95 (0.47)	3.16 (0.36)	70	0.001
Patients receive the best possible help for pain and/or discomfort	3.38 (0.65)	3.70 (0.52)	76	<0.001
Patients receive the best possible help for tiredness (lack of energy)	2.52 (0.70)	2.51(0.78)	75	0.700
Patients receive the best possible help for nausea/vomiting	3.30 (0.77)	3.47(0.55)	76	0.080
Patients receive the best possible help for loss of appetite	2.71 (0.71)	2.69 (0.80)	76	0.994
Patients receive the best possible help for shortness of breath	3.26 (0.70)	3.55(0.55)	76	<0.001
Patients receive the best possible help for depression (feeling sad)	2.51 (0.70)	3.05(0.67)	76	<0.001
Patients receive the best possible help for anxiety (feeling nervous)	2.91 (0.75)	3.39(0.57)	76	<0.001
Patients receive the best possible help for lack of sleep	2.86 (0.74)	2.97(0.65)	77	0.178
Patients receive the best possible help for constipation/diarrhea	3.19 (0.69)	3.09(0.54)	75	0.324
Patients receive the best possible medical care (single item)	3.08 (0.69)	3.38(0.68)	74	<0.001
Patients receive best possible facilitation of personal hygiene (single item)	3.04 (0.68)	3.03(0.64)	74	0.788
**Physical–technical conditions**	2.90(0.53)	3.07 (0.50)	73	0.058
Patients receive help within acceptable waiting time	2.88 (0.73)	3.20(0.64)	73	0.005
Patients receive food and drink that they want	2.87 (0.72)	3.03(0.64)	74	0.236
Patients have access to necessary equipment	2.96 (0.72)	2.99(0.71)	74	0.869
**Identity-oriented approach**	2.96(0.54)	3.08(0.45)	71	0.055
Patients receive useful information on how care and treatment take place	2.83 (0.71)	3.01(0.65)	74	0.056
Patients receive useful information on the effects and use of medicine	2.59 (0.72)	2.57(0.80)	73	0.938
Patients receive useful information on their illness and symptoms	2.84 (0.72)	2.84(0.72)	73	0.949
Patients receive useful information on what they may expect in the near future (development of the illness and symptoms, their health and functioning)	2.87 (0.78)	2.99(0.75)	73	0.169
Patients receive useful information on how to take care of themselves	2.47 (0.76)	2.58(0.78)	72	0.199
The personnel in the team give honest answers to the patient’s questions	3.51 (0.66)	3.51(0.58)	77	1.000
The personnel in the team understand how patients experience their situation	2.96 (0.75)	3.30(0.65)	77	<0.001
The personnel in the team are respectful towards the patient	3.59 (0.62)	3.75(0.44)	76	0.019
Patients have good opportunities to participate in decisions on medical and nursing care	2.95 (0.86)	3.11(0.76)	76	0.076
**Sociocultural atmosphere**	3.04(0.53)	3.27(0.50)	72	<0.001
The personnel in the team support the patient in living their life in a meaningful way	3.01 (2.76)	3.33(0.70)	76	0.003
The personnel in the team support the patient by tending to their spiritual and existential needs (single item)	2.62 (0.79)	3.05(0.85)	73	<0.001
Patients’ relatives are treated with respect	3.55 (0.62)	3.49(0.60)	75	0.372
Patients’ relatives receive the best possible information, support, and care	3.18 (0.74)	3.30(0.61)	76	0.207
Patients usually receive help from the same physician and the same nurse	2.65 (0.82)	2.92(0.87)	77	0.013
Patients’ care is determined by their requests and needs rather than staff procedures	2.76 (0.67)	3.22(0.70)	75	<0.001
The personnel cooperate well	3.10 (0.68)	3.31(0.69)	77	0.010
There is a pleasant and secure atmosphere where the patients receive their medical and nursing care (single item)	3.32 (0.68)	3.50(0.58)	76	0.023

* *p-*values refer to differences in Wilcoxon signed rank test. Statistical significance was set at *p* < 0.05.

### NHPs’ perceptions of the palliative care quality received

On the PR scale, none of the dimensions or single items received a ‘high’ score. At the item level, two items in the identity-oriented approach scored high: ‘The personnel in the team give honest answers to the patient’s questions’ and ‘The personnel in the team are respectful towards the patient’. In the dimension of sociocultural atmosphere, one item scored high: ‘Patients’ relatives are treated with respect’. Three out of four dimensions received a ‘low’ score: medical-technical competence, physical-technical conditions, and identity-oriented approach. One of four single items about tending to patients’ spiritual and existential needs scored low. At the item level, 14 of the 24 items and five of the seven symptom-specific items scored low. These were related to symptom management of tiredness, loss of appetite, depression, anxiety, and lack of sleep. Additionally, low scores were found in items about waiting time, food, equipment, meeting needs for information, patient participation, understanding patients, spiritual and existential care, individualized care, and continuity of care.

### NHPs’ perceptions of what is important for patients with life-threatening illnesses in nursing homes

Subjective importance was not perceived as high at the dimension level, but one single item scored high: ‘There is a pleasant and secure atmosphere’. However, at the item level and for the symptom-specific questions, two items in the medical-technical competence dimension were high: ‘Patients receive the best possible help for pain and/or discomfort’ and ‘Patients receive the best possible help for shortness of breath’. Two items scored high in the identity-oriented approach dimension: ‘The personnel in the team give honest answers to the patient’s questions’ and ‘The personnel in the team are respectful towards the patient’.

Subjective importance was not perceived as low for any of the dimensions or single items. Six items scored low; these were related to access to equipment, information and continuity of care. Additionally, three symptom-specific items had a low score: help for tiredness, lack of appetite, and lack of sleep.

### Comparison of NHPs’ perceptions of the care received and importance of the care areas

In comparing the scores of perceived reality and subjective importance, statistically significant differences were found. When SI was statistically significantly higher than PR, this was interpreted as an area in need of improvement. The SI scores were statistically significantly higher than the PR scores in the two dimensions of medical–technical competence and sociocultural atmosphere, and for three single items: ‘Patients receive the best possible medical care’, ‘The personnel in the team support the patient by tending to their spiritual and existential needs’ and ‘There is a pleasant and secure atmosphere’. For six out of the 24 items and three out of the seven symptom-specific questions, SI also scored statistically significantly higher than the care received. These were related to receiving symptom relief for pain and/or discomfort, shortness of breath, depression and anxiety, in addition to receiving help within acceptable waiting time, understanding how patients experience their situation, respect towards patients, supporting patients in living their lives in a meaningful way, continuity in receiving help from the same physician or nurse, cooperation between personnel and whether care was determined by patients’ requests and needs rather than staff procedures.

The results for the three yes/no items in the identity-oriented approach dimension showed that 79.7% of the NHPs considered that patients had received information about their responsible physician, while 80.6% answered ‘yes’ to the corresponding question about the patients’ responsible nurse. The NHPs also found that patients were given the opportunity to participate in drawing up their individual care plan (80.8%).

## Discussion

The aim of this study was to investigate physicians’ perspectives of palliative care quality in Norwegian nursing homes. The results showed that the NHPs scored the subjective importance statistically significantly higher than perceptions of actual care received for two dimensions, three single items and 13 items.

### NHPs’ perceptions of quality of care and what is important for patients in nursing homes

The results showed that the NHPs perceived care quality (PR scale) to be high for the following; giving honest answers to the patient’s questions and being respectful towards the patient and relatives. However, most dimensions and items scored low on quality of care received, e.g. symptom relief, information, continuity, and individualized/person-centered care. This may indicate that NHPs find the quality of palliative care to be inadequate for NH patients in many care areas. This finding has been confirmed in previous studies on the perspectives of HCP in various care contexts [25,26].

The NHPs in this study found alleviation of pain/discomfort and shortness of breath, honesty and respectful behavior, and a comforting atmosphere in the ward to be of high importance to patients. However, six items related to information and continuity of care and three symptom-specific items (tiredness, poor appetite, and lack of sleep) were considered to be of low importance to the patients. These NHPs’ scores are lower than results from previous studies of the perspectives of HCP [25,26] and from a study of patients’ perspectives in various palliative care contexts ^[^17].

### The services’ ability to meet patients’ care preferences and areas for improvement

The NHPs’ perceptions of care quality can be interpreted as; (1) *Ability of services to meet patients’ care preferences.* These are care aspects with no significant difference between PR and SI, which indicates a strength when similarly high PR and SI scores are present; (2) *Areas for improvement*, which comprise care areas in which the SI score was significantly higher than the PR score, indicating that the care received was perceived as inadequate in relation to what was considered important to patients [17] The results therefore need to be discussed in terms of strengths and areas for improvement.

From the perspectives of NHPs in this study, areas for improvement were related to medical care, relief of symptoms, existential care, atmosphere, waiting time, individual care, cooperation/continuity, understanding how patients experience their situation, respect towards patients and supporting patients in a meaningful way. However, medical care, relief of pain and discomfort, respect towards patients and atmosphere in the ward had relatively high scores on both PR and SI, which might suggest that these specific areas were perceived as especially important for patients. The ability of the NHs to meet patients’ care preferences in this study, seemed to be particularly related to giving patients honest answers and treating patients’ relatives with respect. These results mainly concur with previous studies of the perspectives of HCP and patients [17,25,26] However, those studies identified information to patients as an area for improvement, particularly information about care and treatment, medications, prognosis, illness, and symptoms. Surprisingly, this was not identified in the present study. This might be understood in light of the NH context where patients receive long-term care and, to a lesser extent, active treatment [4]. Further, many have cognitive impairment, which can make it challenging to provide detailed information about medications and illness. Nevertheless, a previous study showed that physicians regarded the provision of information as an important medical task in nursing homes, ^4^ which to some extent contradicts the results in this study. This finding may also be influenced by the NHPs’ own interpretation of what is important, as opposed to evaluating the care on behalf of the patients. This underlines the need for careful instructions when using proxies to evaluate care quality.

The results of this study call for improvement of palliative care in the NH context. In Norway, national guidelines have been developed; strategies for enhancing HCP competence in palliative care, including in NHs, have been described in government documents [5,7]. However, the results of this study indicate incongruence between guidelines and the reality. Froggatt et al. have suggested a framework to implement palliative care in long-term facilities [31]. This framework consists of recommendations for implementation of the principles of palliative care within the organization (micro level), across organizations (meso level) and at national or regional level (macro level). The framework emphasizes staff competence and an ongoing learning culture, team approach, support for managers in introducing the implementation of palliative care principles and regulatory framework and legislation to promote palliative care provision in long-term care facilities [31]. This framework might give useful guidance for further improvement initiatives related to the results of this study.

### Methodological considerations

One limitation of this study is related to the low number of respondents and the low response rate of 43.5%, which weakens the statistical power and makes it difficult to generalize the results. Despite detailed information about the study and multiple reminders by mail to the recruiting personnel, the final number of respondents was low. The low response rate suggests that physicians in NHs may be difficult to recruit due to their very heavy workload. Further, in Norway there is a limited number of physicians working in the NH context. Low response rates have been previously described to be challenging and are often well below 50% [32]. However, this study recruited respondents from densely and sparsely populated areas in all the health regions of Norway, which is considered to be a strength of the study. Due to the relatively small sample size and lack of normal data distribution, we performed a non-parametric statistical analysis [30].

The QPP-PC has previously been psychometrically evaluated in patient samples [16,22]. The instrument used in the present study is adapted from the patient version. The adaptation consisted of changes of wording from a patient’s perspective to suit NHPs’ perceptions of the patient’s perspective. NHPs’ perspectives cannot fully replace the patient perspective. However, there are both strengths and limitations related to assessment of care on behalf of the patient. On one hand, the care might be assessed differently by patients and physicians, which can lead to over- or underestimating the care quality and the importance of the care aspects, compared to the perspectives of the patients [33,34]. On the other hand, this method can give voice to patients that are unable to communicate their views about the care they receive and the importance of this care, due to advanced illness and/or cognitive impairment. Many patients within this category are admitted to NHs. The adapted version has not yet been evaluated in relation to HCP/NHPs’ perspectives on behalf of patients. However, it has been used in similar samples [25,26]. The NHPs scored the SI of some items (e.g. information about medication, help for tiredness) at below 3.0, which could be interpreted as of low importance. This could indicate that NHPs evaluate the items in the QPP as less important for this specific patient group, which could compromise the validity of the instrument. However, previous findings in studies of the perspectives of HCP in various contexts [25,26] and patient perspectives in various contexts including NHs [17,35] have shown that these items were scored as important to very important. The internal consistency of the QPP-PC showed acceptable α values (>0.7) for all dimensions in the two scales, except for the PT dimension where alpha levels were 0.55 and 0.64 for the PR and SI scale respectively, which can be explained by the low number of items in this dimension. This is in line with previous studies using this instrument with HCP and patient respondents [22,25].

## Conclusion

This study provides knowledge of how NHPs perceive palliative care quality in Norwegian nursing homes. The results show that provision of high-quality palliative care in nursing homes can be challenging and the NHPs identified several areas for improvement. A need for improvement was identified in care areas such as symptom relief, existential care, waiting time, individualized care, cooperation, and continuity. The results can provide direction for future quality improvement initiatives, particularly in terms of systematic implementation of palliative care principles in NH contexts by prioritizing training and competence development. However, these findings need to be confirmed in a larger sample.

## Data Availability

The data used and analyzed in the present study are not publicly available and cannot be shared due to the data protection regulations and the informed consent from respondents. Access to data is limited to the researchers, who have obtained permission for data processing from Norwegian Agency for Shared Services in Education and Research (ID: 777028), the local research ethics committee at the University of Inland Norway (Ref. No.: 23915412).
